# Breast Feeding Practice: Positioning and Attachment during Breast Feeding among Lactating Mothers Visiting Health Facility in Areka Town, Southern Ethiopia

**DOI:** 10.1155/2019/8969432

**Published:** 2019-04-07

**Authors:** Nega Degefa, Befikadu Tariku, Takalign Bancha, Gizachew Amana, Abdo Hajo, Yisehak Kusse, Eshetu Zerihun, Zeleke Aschalew

**Affiliations:** ^1^Nursing Department, College of Medicine and Health Sciences, Arba Minch University, Arba Minch, Ethiopia; ^2^Public Health Department, College of Medicine and Health Sciences, Arba Minch University, Arba Minch, Ethiopia; ^3^Nursing Department Students, College of Medicine and Health Sciences, Arba Minch University, Arba Minch, Ethiopia

## Abstract

**Background:**

Breastfeeding is the act of milk conveyance from the mother to the infant. Adequate nutrition during infancy and early childhood are mandatory to ensure growth, health, and development of children to their maximum potential. The positioning of the baby's body is important for maintaining good attachment and successful breastfeeding. Most difficulties of breastfeeding can be avoided altogether if good attachment and positioning can be achieved at the first and early feeds. Plenty of studies have been conducted to explore factors affecting breastfeeding practice in general; however, there is a meagerness of evidence that assesses factors affecting attachment and positioning during breastfeeding specifically. Therefore, the current study was aimed to assess positioning and attachment during breastfeeding among lactating mothers visiting health facilities of Areka town.

**Methods:**

an institution-based cross-sectional study was conducted by using observational checklist adopted from the World Health Organization breastfeeding observation form. Maternal-related characteristics were collected by using a structured and pretested questionnaire. The study was conducted from April to June 2017. Respondents were selected by using a systematic random sampling technique. Descriptive summaries were done to present pertinent findings and a chi-square test was used to check association between variables.

**Result:**

There was poorer positioning among primipara mothers (47.1%) than multipara mothers (28.7%). A poor attachment was also more apparent among primipara mothers which were more (31.1%) than the multipara (27.1%) mothers.

**Conclusion:**

Younger mothers (<20 years old), the primipara, and those who have no formal education deserve more attention, support, and direction to make sure that they can achieve proper positioning and attachment during breastfeeding at the first and early feeds.

## 1. Introduction

Breastfeeding is the act of milk transference from the mother to the infant [1]. Adequate nutrition during infancy and early childhood is essential to ensure the growth, health, and development of children to their maximum potential [2]. Early initiation of breastfeeding, within one hour of birth, protects the newborn from acquiring infection and reduces newborn mortality [3, 4]. According to global public health recommendation, infants should be exclusively breastfed for the first six months of life to achieve optimal growth, development, and health [2, 5–7]. Thereafter, to meet their evolving nutritional requirements, infants should receive nutritionally adequate and safe complementary foods while breastfeeding continues up to two years or beyond [2, 6, 8, 9].

The positioning of the baby's body is important for good attachment and successful breastfeeding. Most difficulties of breastfeeding can be avoided altogether if good attachment and positioning can be achieved at the first and early feeds. The word “attachment” describes how the baby's mouth takes the breast and “positioning” describes how the baby's body is put near the mother's body [10].

In Africa, Asia, Latin America, and the Caribbean, only 47-57% of infants less than two months and 25-31% of infants 2-5 months are exclusively breastfed, and the proportion of infants 6-11 months of age receiving any breastmilk is even lower [11]. Suboptimal breastfeeding, especially nonexclusive breastfeeding in the first 6 months of life, results in 1.4 million deaths and 10% of the disease burden in children younger than 5 years [11, 12].

In Ethiopia, data from 2016 Ethiopian demographic and health surveillance (EDHS) showed that, overall, 58% of children under age of 6 months are exclusively breastfed, and the percentage of exclusive breastfeeding declines with age from 74% in 0-1 months to 36% in 4-5 months [13].

Multitudes of factors can influence the patterns and early initiation of exclusive breastfeeding to infants. Based on the reports of a study done at Ofa district, maternal education, husbands education, antenatal care, postnatal care, and colostrum feeding positively affected the initiation of breastfeeding and exclusive breastfeeding [14].

Plenty of studies explore factors affecting breastfeeding practice in general; however, there is a meagerness of studies that assess factors affecting attachment and positioning during breastfeeding specifically. Therefore, this study was aimed to assess factors associated with attachment and positioning during breastfeeding among lactating mothers visiting postnatal and immunization units of health facilities in Areka town.

## 2. Method and Materials

### 2.1. Study Area and Period

The study was conducted at health facilities found in Areka town. The town is one of the city administrations of Wolayita Zone and found in North West of Sodo, capital of the zone at 29km. The town is located at 300km from Addis Ababa. The study was conducted from April to June 2017.

### 2.2. Study Design

An institution-based cross-sectional study was employed among mothers who visited the postnatal and immunization unit of health facilities in Areka town.

### 2.3. Population

The source population was all mothers who visited the postnatal and immunization unit at Areka Health Center and Dubbo Hospital, whereas the study population was all sampled lactating mother-infant-pairs who visited the postnatal and immunization unit of Areka Health Center and Dubbo Hospital and satisfying the inclusion criteria. All mothers who visited the postnatal and immunization unit of Dubbo Hospital and Areka Health Center during the study period were included in the study, whereas mothers who had serious illnesses and/or neonatal malformations of palate/tongue were excluded from the study.

### 2.4. Sample Size Determination and Sampling Procedure

The sample size was calculated by using single population proportion formula by considering the following assumptions: A 95% confidence level, a margin of error 5 %, and 10% for nonresponse and the proportion of breastfeeding practices was 78% from the study conducted in Ofa district [14]. Finally, 290 mother-infant pairs were sampled for interview and observation during breastfeeding.

To select the respondents, we first identify the average number of mothers who visited the postnatal and immunization unit of Areka Health Center and Dubbo Hospital daily for the last two months from the client registration book/record. Depending on the predetermined client flow rate the average number of client that could be expected during the study period was estimated. Then, the total sample was proportionally allocated for both facilities. Finally, participants were selected using a systematic random sampling technique. The sampling interval was calculated by dividing the expected number of the client during the study period for the allocated sample of each health facility.

The following criterion for scoring and grading of correct positioning and child's mouth attachment to the breast was developed and adopted based on WHO criteria [15].

*Correct positioning*: criteria for correct body positioning comprises: mother relaxed and comfortable, mother sit straight and well-supported back, trunk facing forward and lap flat, baby neck straight or bent slightly back and body straight, the baby's body turned toward the mother, the baby's body close to mother body and facing breast, and the baby's whole body supported.

*Grading and soring of correct positioning:* if one criterion from the mother's position and one criterion from the infant's position or both from mother's position was achieved, it was graded as poor and scored 1-2, if at least one criteria from the mother's position and two or three criteria from the infant's position were achieved, it was graded as average and scored 3-4 and if at least two criteria from the mother's position and three or four criteria from the infant's position or all criteria was achieved it was graded as good and scored 5-7.

*Correct attachment:* criteria for correct attachment of infants mouth to the breast incorporate chin touching the breast, mouth wide and open, and lower lip turned outward, and more areola is seen above the baby's mouth.

*Grading and soring of correct attachment:* if any one of the four criteria was achieved, it was graded as poor and scored 1, if any two of the four criteria were attained it was graded as average and scored 2, and if any three or all the four criteria were attained it was graded as good and scored 3-4.

### 2.5. Data Collection

Data were collected through observation and client exit interview. Observational breastfeeding checklist adopted from WHO was used for observation. The mother was asked to put her infant to the breast and observed for breastfeeding process by five trained nursing students, for five minutes and recorded the mother and infant's positioning and attachment to the breast according to WHO beast feed observation form.

A structured and pretested questionnaire was developed by reviewing prior studies done on the same topic. This was primarily used to collect maternal-related characteristics like age, occupation, level of education, parity, marital status, husband's level of education, and gestational age.

### 2.6. Data Processing and Analysis

The consistency and completeness of the collected data were checked manually and then coded, cleaned, and entered by using Epidata version 3.1 and then exported to statistical package for social sciences (SPSS) version 20 software for further analysis. Descriptive statistics like frequencies, percentage, proportions, and summary statistics were used to define respondents in relation to pertinent variables and presented by using tables. The association between variables was measured by using a chi-square test. P value < 0.05 was considered significant in all cases.

### 2.7. Data Quality Assurance

A standardized observational checklist adopted from WHO breastfeed observation form [15] was translated to local language and the responses were retranslated to English for consistency. A pretest was done on 5% of the sample and the result was used to correct wording, spelling, and approach of the questionnaire. Training was given for data collectors on the content of the questionnaire and observational checklist for two consecutive days.

### 2.8. Ethical Considerations

An official letter from the Department of Nursing, College of Medicine and Health Sciences, Arba Minch University, was submitted to all concerned bodies to ensure their cooperation. Informed verbal consent was obtained from each participant before the actual data collection.

## 3. Result

### 3.1. Sociodemographic Characteristics of the Mother

A total of 252 mothers participated in the study giving a response rate of 87%. The mean age of the mothers was 28±5.32 years. Of the total participants, 11 (4.36%) were aged less than or equal to 19 years and 22 (8.73%) were aged 36 and above. Slightly more than half (52.8%) of all mothers were from the urban area. Most of the respondents, 231(91.7%), were married. Among the total respondents, 132(52.4%) were protestant religion followers. Ninety percent of respondents in the study area were Wolayita in an ethnic group. Eighty-six percent of the respondents had attended no formal education. Out of 252 mothers, 142(56.3%) were housewives (Table 1).

### 3.2. Infant-Related Characteristics

Out of the total infants, 10 was born preterm, 211 born at term, and 14 born postterm. From the total respondents, nearly two-fifths (43.3%) of mothers knew the exact weight of their child at birth and the rest (56.7%) do not remember, but simply guess the weight of their child at birth by saying big, medium, and small. Out of 109 infants with known weight at birth 95(87.2%) had normal birth weight (>2.5Kg).

### 3.3. Maternal Healthcare Service Utilization Related Characteristics

The current study indicates that, 187 (74.2%) and 97 (38.49%) of all mothers had attended antenatal and postnatal care, respectively. Out of 187 mothers who had antenatal care follow-up, 118 (63.1%) had four or more visits and 128 (68.45%) received information on breastfeeding. A total of 199 (79%) of all mothers delivered their index child in health facilities. From them, nearly 10% (23) gave birth through cesarean section and the remaining by spontaneous vaginal delivery. Among fifty-three mothers who delivered at home, most of them (88.67%) were assisted by traditional birth attendants and only 6 (11.33%) were assisted by health care professionals.

### 3.4. The Position of Mother and Infant and Attachment to the Breast during Breastfeeding

Proper positioning of mother and infant during breastfeeding was poorer among 38.1% of respondents. About thirty-six percent and 25.8% of all mothers had average and good positioning during breastfeeding, respectively. The chi-square test showed that parity and residency have an association with positioning during breastfeeding. Attachment of baby (latch-on) to the breast was associated with both maternal and neonatal factors. Place of delivery was one of the factors associated with the attachment of the baby during breastfeeding (Table 2)

### 3.5. Infant-Related Factors Associated with Attachment (Latch-On)

Attachment (latch-on) to mother's breast was poorer among preterm babies (50%) when compared with full-term babies (29.4%). There is also poorer attachment among low birth weight babies which is 64.3% when compared to their counterpart (Table 3).

## 4. Discussion

The finding of this study suggests that mothers who are aged between 20 and 30 years had better positioning and attachment while breastfeeding than those who are aged less than 20 years old. The older the mother's age gets, the more they have experience and knowledge about breastfeeding. But age was not associated under chi-square test with position and attachment. Studies conducted by, Goyal RC (Libya), Santo et al. from Brazil and Gupta et al. (north India) reported similar findings [16–18].

Our study revealed that 44.2% of the mothers who have attended no formal education have poorer positioning while breastfeeding. Mothers with a higher level of education are more likely to look for health information and implement it properly. However educational level was not significantly associated with either attachment/positioning. Unlike our finding a study by Kronborg H and Væth M mentioned that mothers with a higher level of education were more likely to apply proper skills of breastfeeding [19]

In this study primipara, mothers (woman giving birth for the first time) had poorer positioning and attachment while breastfeeding when compared to their counter multipara mothers. This could be basically the result of knowledge and experience the mothers may attain previously. Goya RC et al. reported a similar finding [16], whereas Gupta et al. from north India mentioned no statistically significant association between parity and positioning and attachment [18]

The current study indicates that preterm (born before 37 weeks of gestation) infants had poorer attachment. The smaller the age of the newborn, the less mature physical and physiologic function they will have, which, in turn, hinders the babies ability to attach to the breast properly. Goya RC et al. reported* s*imilar finding [16]. However, a study by Coca et al. revealed no significant association between gestational age and effective suckling and attachment [20].

## 5. Conclusion and Recommendation

Mothers below the age of twenty years, primipara mothers, and those who had no formal education deserve more attention, support, and direction to make sure that they can effectively breastfeed their baby.

Healthcare facilities should stress the need for updated training for healthcare providers to improve their skills of providing breastfeeding counseling and education during antenatal and postnatal follow-up.

It would also be better if mothers are demonstrated with skills of proper attachment and positioning then observed by the healthcare provider while they breastfeed their baby on immediate postnatal care and given feedback on their performance to achieve proper attachment and positioning.

Mothers should be encouraged by family and community health worker to plan to have trained birth attendant and give birth in a health institution. If birth is at home, the first postnatal contact should not be delayed more than 24 hours or other skilled providers and/or well-trained, community health worker should supervise.

## Figures and Tables

**Table 1:** Sociodemographic characteristics of mothers visiting health facilities in Areka town, Southern Ethiopia, 2017.

Variable	Category	Frequency (%)
Marital status	Never married	2(0.8 )
	Married or living together	231(91.7)
	Divorced/separated/widowed	19(7.5 )

Husband's educational level	No formal education	83(32.9)
	Primary	72(28.6)
	Secondary	56(22.2)
	Above secondary	41(16.3)

Age of respondents	<20	21(8.3)
	20-30	174(69.0)
	>30	57(22.6)

The religion of the respondent	Protestant	132(52.4)
	Orthodox	94(37.3)
	Catholic	16(6.3)
	Muslim	10(4)

Ethnicity	Wolayita	224(88.9)
	Gurage	13(5.2)
	Amhara	8(3.2)
	Hadiya	7(2.8)

Mothers educational level	No formal education	86(34.1)
	Primary	84(33.3)
	Secondary	52(20.6)
	Above secondary	30(11.9)

**Table 2:** Cross-tabulation of maternal characteristics and position of mother and infant and attachment (latch-on) to the breast during feeding among mothers visiting health facilities in Areka town, Southern Ethiopia, 2017.

Characteristics	Grade of positioning			Attachment grade		
	Poor N (%)	Average N (%)	Good N (%)	Poor N (%)	Average N (%)	Good N (%)
*Maternal age *						
<20[n=21(8.3%)	10(47.6)	8(38.1)	3(14.3)	9(42.9)	6(28.6)	6(28.6)
20-30[n=174(69%)	64(36.8)	60(34.5)	50(28.7)	48(27.6)	66(34.5)	60(34.5)
>30[n=57(22.6%)	22(38.6)	23(40.4)	12(21.1)	18(31.6)	24(26.3)	15(26.3)
	*x* ^*2*^ *=3.176, p=0.529*			*x* ^*2*^ *=3.274, p=0.513*		

*Maternal education *						
No formal education	38(44.2)	30(34.9)	18(20.9)	26(30.2)	37(43.0)	23(26.7)
Primary	31(36.9)	29(34.5)	24(28.6)	29(34.5)	30(35.7)	25(29.8)
Secondary	15(28.8)	22(42.3)	15(28.8)	13(25.0)	19(36.5)	20(38.5)
> secondary	12(40.0)	10(33.3)	8(26.7)	7(23.3)	10(33.3)	13(43.3)
	*x* ^*2*^ *=4.013, p=.675*			*x* ^*2*^ *=5.087, p=.533*		

*Parity *						
1	56(47.1)	37(31.1)	26(21.8)	37(31.1)	43(36.1)	39(32.8)
2-5	37(28.7)	53(41.1)	39(30.2)	35(27.1)	52(40.3)	42(32.6)
>5	3(75.0)	1(25.0)	0(0.0)	3(75.0)	1(25.0)	0(0.0)
	*x* ^*2*^ *=12.354, p=0.015∗*			*x* ^*2*^ *=4.895, p=0.298*		

*Residence *						
Rural	55(39.9)	41(29.7)	42(30.4)	38(27.5)	49(35.5)	51(37.0)
urban	41(36.0)	50(43.9)	23(20.2)	37(32.5)	47(41.2)	30(26.3)
	*x* ^*2*^ *=6.257, p=0.044∗*			*x* ^*2*^ *=3.243, p=0.198*		

*Place of delivery *						
Home	17(32.1)	24(45.3)	12(22.6)	14(26.4)	30(56.6)	9(17)
Health facility	79(39.7)	67(33.7)	53(26.6)	61(30.7)	66(32.2)	72(36.2)
	*x* ^*2*^ *=2.460, p=0.292*			*∗x* ^*2*^ *=11.088, p=0.004*		

*n*=number of mothers; *∗* significant *at* p< 0.05.

**Table 3:** Cross-tabulation of Attachment (latch-on) and infant characteristics at Areka town, Southern Ethiopia, 2017.

Variable	Grade of attachment		
	Poor N (%)	average N (%)	Good N (%)
*Gestational age *			
Preterm	5(50)	3(30)	2(20)
Full term	62(29.4)	84(39.8)	65(30.8)
Post-term	8(25.8)	9(29)	14(45.2)
	*x* ^*2*^ *=4.492,p=0.343*		

*Birth weight *			
<2.5kg	9(64.3)	3(21.4)	2(14.3)
≥2.5 kg	27(28.4)	30(31.6)	38(40.0%)
	*x* ^*2*^ *=7.102, p=0.029∗*		

*∗*Significant *at* p< 0.05.

## Data Availability

You can have the dataset used and/or analyzed during the current study from the corresponding author on a reasonable request.

## Abbreviations

ANC: Antenatal care
PNC: Postnatal care
EDHS: Ethiopian Demographic and Health Survey
WHO: World Health Organization
AOR: Adjusted odds ratio
CI: Confidence interval
MCH: Maternal and Child Health
WHO: World health organization.
